# Racing for a SARS‐CoV‐2 vaccine

**DOI:** 10.15252/emmm.202115145

**Published:** 2021-09-27

**Authors:** Özlem Türeci, Uğur Şahin

**Affiliations:** ^1^ BioNTech SE Mainz Germany; ^2^ University Medical Center of Johannes Gutenberg University Mainz Germany

**Keywords:** Immunology

## Abstract

Interview with EMBO Members Özlem Türeci and Uğur Şahin, BioNTech, conducted by science journalist Kai Kupferschmidt.

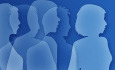

*Interview with EMBO Members Özlem Türeci and Uğur Şahin, BioNTech*. *Conducted by science journalist Kai Kupferschmidt, Berlin*.

**Kai Kupferschmidt (KK):** When you founded BioNTech in 2008 after you had founded GANYMED Pharmaceuticals in 2001, what was the idea behind that new company?

**Uğur Şahin (US):** Our motivation as immunologists has always been to help patients—originally patients with cancer. One of the key challenges here is that each patient’s tumor is different. When comparing tumors of two patients with the same type of cancer, the similarity of their tumors is less than 3% and more than 97% is unique. The variability and heterogeneity of human cancers is the root cause for the failure of many cancer therapies. We wanted to find better ways to address this challenge and treat patients with cancer. For us, the solution is individualized medicine. Our vision is to address the uniqueness of each patient’s tumor and to develop novel technologies that enable individualized treatments. This is exactly why we have founded BioNTech, which stands for **Bio**pharmaceutical **N**ew **Tech**nologies.

**KK:** At what point did mRNA technology become the focus?

**Özlem Türeci (ÖT):** I would rather say that mRNA is one of our focuses. As scientist, we are solution‐ and goal‐driven: Our goal is to provide better medicines for patients by harnessing the full potential of the immune system. mRNA has been one of four technologies, which we started to adapt in the early 2000s for different therapeutic modalities such as vaccines, but also for expressing antibodies or cytokines. Besides antibodies, cell therapies, and small molecules, mRNA is our most advanced technology. We have developed and optimized our technology over more than two decades for use. When we were hit by the pandemic, we had just matured it to the right level to use it as a vaccine technology. The COVID‐19 vaccine development was baptism by fire. It was a proof concepts highlighting the potential of this new drug class.

**KK:** At what point did you look at your mRNA technology and say, this is now ready, we can use this?

**ÖT:** The starting point in time lies back in our research time on mRNA technology even before BioNTech, which we started in the late 1990s. At that time mRNA was overlooked, because it was not potent enough: Once mRNA was brought into a cell, its half‐life and the translation efficiency were so low that the amounts of protein would not be sufficient. We invested several years to develop our mRNA technology by modifying the non‐coding regions of the mRNA, the cap region, the UTRs, the poly‐T region, how to purify it, and how to get the pharmacokinetics and the translational efficiency right. By combining better designs of those elements, we achieved a more than 1,000‐fold increase in antigen yield. Moreover, we discovered strategies to deliver mRNA into dendritic cells in lymphoid tissues which dramatically improved immune responses. In 2014, the intravenous lipid mRNA was the first one we developed into clinical stage for our cancer vaccines. In the same year, we started the first individualized mRNA cancer vaccine trial. Both studies documented strong mRNA vaccine‐induced immune responses and provided highly encouraging clinical results. This proof of concept was in 2017 that means more than 20 years after we started our research and motivated us to initiate a number of late‐stage clinical trials, which are currently ongoing. Another improvement which benefits our mRNA toolbox is the nucleoside modification, which was developed by Katalin Karikó and Drew Weissman, which blunts the immune modulatory feature and ensures that mRNA is translated longer.

**KK:** Can you take me back to the beginning of 2020 when an article in *The Lancet* in early January reported the first cases of SARS‐CoV‐2 infections in China?

**US:** In January 2020, I read the publication in The Lancet that described the first cases of SARS‐CoV‐2 infections in Wuhan which displayed the full pattern of a pandemic threat: There were individuals who did not have symptoms, no fever, even though they were positive for the virus. In addition, there were no effective traveling restrictions for people at that time. In our global world, it was very clear to me that the virus causing this outbreak had already spread worldwide that means we were already in a pre‐pandemic phase and we had to act fast. I convinced Özlem and then the executive and supervisory team and together we decided to contribute with our technology in order to help develop a vaccine against the virus as fast as possible.

**KK:** I'm really struck that this was in January when you had this realization. What did you see that other people missed? What did you put in a position to make that call?

**US:** First of all, I think many people, not only I and our team, saw that, but what limits us as human beings is the past experience. A pandemic threat in this dimension did not happen in the past 50 years. It was a new situation with a new pathogen, which fulfilled the full pattern of a global outbreak. In addition, the combination of high infectivity, a more or less immune naive human population, a higher transmission rate and the pathology made it nearly impossible to control during that early stage. The mathematical evidence was there, and we decided early to act fast and to not lose time.

**KK:** What were the kinds of decisions that you made at the company? What changed in January 2020?

**ÖT:** At that weekend when took this decision, we had already considered to design more than 20 vaccine candidates and the cloning of multiple constructs was initiated. In fact, we did many steps in parallel to save time without taking any shortcuts. Our teams worked in 24/7 shifts. Everything was initiated and put on tracks and then escalated and accelerated over time with more information on the reality of the pandemic coming in. In addition, we early started the dialogue with regulatory authorities and the Federal Institute for Vaccines and Biomedicines, the Paul‐Ehrlich‐Institute (PEI). It was a race against time. Everyone involved pulled toward the same goal: develop a safe and effective vaccine as fast as possible.

**KK:** The first person was vaccinated at the end of 2020. We are now in a situation where we are vaccinating teenagers in Israel, Germany and the United States. And then there are other places in the world where there is no vaccine even for frontline health care workers. How do you think about this, and where is your responsibility in a sense?

**ÖT:** The question is after equality of distribution and that has been important for us from the very beginning. No one is safe, until everyone is safe. We are already working on sustainable solutions to foster a broader, a global supply, and we decided to execute on a three‐step approach:

- **As a first action,** we signed the COVAX agreement to deliver 40 million doses at a not‐for‐profit price to low‐ and lower‐middle‐income countries.
- **In parallel**, we have increased our manufacturing capacity and decided to deliver 2 billion doses of our COVID‐19 vaccine to low‐ and middle‐income countries until end of 2022. The first 1.2 billion doses will be provided this year. This equals 40% of the manufacturing capacity of ours and Pfizer’s network.

**US:** Besides delivering vaccines to low‐income countries, we believe it is important to enable that qualified regions on the African continent and on other regions have access to the technology. We have started the process to identify partners to whom we can transfer our technology and build manufacturing capacity to ensure that low‐ and middle‐income countries have everything in place to manufacture vaccines on their own. In the mid‐term to long term, it is our goal to develop sustainable solutions and to establish a worldwide manufacturing network which is not only suitable to address COVID‐19 but also to address potential future pandemics and other regional health threats. On the African continent, for example, we are currently exploring possibilities to set up state‐of‐the‐art mRNA manufacturing facilities.

**KK:** You have had this fast, amazing success with this vaccine. And that, of course, stands in stark contrast to how hard it has been to develop anything in the cancer field. Is there anything you can take from this experience that will help you with your original mission of cancer treatments?

**ÖT:** Yes, definitely. We were using our experience in cancer vaccines to cross‐fertilize the COVID‐19 development, and what we have learned is now being applied back to cancer. It is not only about what we have learnt, but also about, for example, regulators learning more about the technology and safety profiles, and about learning how to upscale manufacturing and about immune response patterns. This will be a tailwind for our developments in the field cancer medicine and how we will modify, improve, and accelerate our efforts to develop cancer vaccines.

**KK:** Where do you see, in terms of infectious disease, advances that could be made with mRNA that may be low‐hanging fruit?

**US:** What drives us is medical need, and there are a few pathogens for which there is a huge medical need, for example, tuberculosis. We started almost one and a half years ago a project to develop a tuberculosis vaccine in partnership with the Gates Foundation, and we plan to bring vaccine candidates into clinical testing in 2022. The other great challenge is malaria. With the “eradicateMalaria” initiative, led by the kENUP Foundation and their outstanding supporting partner network, we will do whatever it takes to develop a safe and effective mRNA‐based Malaria vaccine. Of course, there are a number of other infectious diseases with great medical need, and we are evaluating whether this is something we would do ourselves or whether we would do it in partnership with other pharma companies.

**KK:** In your construct, you used the two proline residues that Barney Graham found to be stabilizing the pre‐fusion spike confirmation. I am just curious how you made these decisions. Did you read the papers and immediately realize, okay, this could help us?

**US:** The weekend after reading *The Lancet* paper, we took a closer look at about 100 publications with potential relevance to vaccine design, and the data that Barney had described for the SARS virus plus the crystal structure that was published, convinced us that this is a good way to go.

**KK:** The other thing is the nanoparticle technology. What have been the improvements there?

**US:** In principle, this is similar to the development of our mRNA technology: You have to combine many innovations and improvements. In terms of the nanoparticle technology, it is important to ensure protection of the mRNA while ensuring an effective delivery to the right cells in the body. We had tested hundreds of formulations and decided to go with lipids identified by our partner Acuitas, which were already mature for clinical use. We found that their lipids enable the creation of nanoparticles that fulfilled exactly the mode of action in which we were interested: a delivery mechanism to bring the mRNA to lymph nodes where immune responses are primed.

**KK:** Özlem Türeci and Uğur Şahin, thank you for the interview.

*The interview was edited for length and style by Holger Breithaupt and Astrid Gall.*

## Conflict of interest

Prof. Ugur Sahin, M.D., is Co‐Founder and CEO of BioNTech. Özlem Türeci, M.D., is Co‐Founder and Chief Medical Officer of BioNTech.

